# Healthy lifestyle culture in universities as institutional normativity: a sociological systematic review

**DOI:** 10.3389/fsoc.2026.1820442

**Published:** 2026-09-07

**Authors:** Zhaxylyk Tokbergenov, Zhomart Simtikov, Dossym Seitzhanov, Azat Abdrassilov

**Affiliations:** 1 Department of Politology and Socio-Philosophical Disciplines, Abai Kazakh National Pedagogical University, Almaty, Kazakhstan; 2 Department of Initial Military Training, M.Kh. Dulaty Taraz University, Taraz, Kazakhstan; 3 Department of Vocational Training and Fine Arts, O. Zhanibekov South Kazakhstan Pedagogical University, Shymkent, Kazakhstan

**Keywords:** health lifestyles, higher education, institutional normativity, social norms, sociology of education, student wellbeing

## Abstract

**Background:**

Student wellbeing has become an important focus of higher education research; however, existing studies remain largely behavior-oriented and provide limited explanation of how health-related practices are socially produced within university environments. This study aimed to examine how institutional normativity, social norms, and organizational contexts shape healthy lifestyle practices among university students from a sociological perspective.

**Methods:**

A PRISMA-informed systematic review was conducted using Scopus, Web of Science Core Collection, Sociological Abstracts, and Google Scholar to identify relevant studies published between 2010 and 2025. Following a structured selection process, 64 studies were included in the final thematic synthesis.

**Results:**

The findings indicate that students’ practices related to sleep, stress management, physical activity, help-seeking, and substance use are socially organized rather than purely individual behaviors. Universities shape these practices by establishing cultural expectations, institutional routines, and organizational conditions that normalize productivity, digital over-availability, and chronic stress while simultaneously influencing unequal opportunities to maintain healthy lifestyles across different student groups.

**Conclusion:**

The review demonstrates that student wellbeing is embedded within broader processes of institutional governance, social stratification, and everyday practice rather than individual responsibility alone. By integrating fragmented evidence into a coherent sociological framework, this review advances understanding of universities as active producers of student health practices and highlights the importance of institutional approaches for promoting sustainable student wellbeing.

## 1 Introduction

University years represent a pivotal life-course transition in which everyday routines are disrupted and reorganized under new academic demands, peer networks, and institutional expectations. This transition is consistently associated with changes in physical activity, sleep, dietary practices, and substance use—yet these patterns are not simply the outcome of individual “choices.” Rather, they reflect socially organized practices that are embedded in relationships, meanings, and constraints. Evidence syntheses indicate that even when students recognize the value of health-promoting behaviors, participation is strongly shaped by time scarcity, social influences, and the material conditions of university life, including access to facilities, schedules, and affordability (Brown et al., 2024). In parallel, research on campus settings demonstrates that the built and natural university environment is measurably associated with student health-related outcomes, including physical activity and mental well-being, highlighting that health practices are situated within institutional and spatial contexts rather than occurring in a social vacuum (Ding et al., 2024). Within the framework of Education for Sustainable Development, universities are increasingly expected not only to transmit knowledge but also to cultivate competencies that enable students to sustain learning, wellbeing, and responsible participation in society over time. In this sense, healthy lifestyle practices can be interpreted as part of a broader set of transferable competencies—self-regulation, health literacy, stress management, and help-seeking—whose development supports both individual wellbeing and institutional sustainability. However, treating healthy lifestyle culture as a competence-related educational outcome requires attention to the social mechanisms through which such capacities are formed, supported, or undermined in university life.

A sociological approach conceptualizes “healthy lifestyle practices” as elements of broader health lifestyles: patterned, collectively shaped ways of living that emerge at the intersection of structural position, cultural repertoires, and socialization processes. Contemporary scholarship emphasizes that health lifestyles research must move beyond listing behaviors toward explaining how social stratification, institutions, and cultural norms generate durable patterns of practice, while also accounting for change across transitions such as entry into higher education (Mollborn et al., 2021). For students, this is especially relevant because lifestyle formation occurs within dense peer ecologies and normative climates that intensify conformity pressures and stabilize shared understandings of what is “normal,” “acceptable,” and “valued” in student life. From a competence perspective, these normative climates matter because they shape whether health-supportive routines become legitimate, repeatable, and learnable practices within everyday academic participation.

Among sociocultural determinants, social norms occupy a central explanatory position. Norms operate through at least two interrelated mechanisms:

(i) Descriptive norms (perceptions of what most peers do) and
(ii) Injunctive norms (perceptions of what peers approve of).

Norm misperceptions—particularly overestimations of unhealthy peer behaviors—are repeatedly implicated in student risk practices, most clearly in alcohol use. Quasi-experimental evidence from a social norms campaign among Flemish university students suggests that correcting perceived norms can reduce students’ overestimation of peers’ drinking, even when short-term changes in consumption are not immediately detectable (Van Roozendaal et al., 2024). Longitudinal research further indicates that perceived peer drinking norms shift during the transition from high school to college and that stable exposure to high-norm peer contexts is associated with less favorable developmental outcomes, underscoring the role of peer selection and social integration processes in shaping norm trajectories (Benner et al., 2024). These findings reinforce a sociological point that is also crucial for competency-oriented education: norms are not simply “beliefs” within individuals; they are produced and reproduced within peer groups, organizations, and institutional cultures, thereby influencing what practices students can realistically sustain as part of their everyday competence repertoire.

Social norms structure health-related practices beyond substance use, including physical activity and help-seeking. A theory-informed systematic review mapping barriers and facilitators to student physical activity found “social influences” to be among the most consistently salient domains across qualitative and quantitative studies, alongside environmental resources and competing goals (Brown et al., 2024). This convergence suggests that physical activity in university populations is shaped by social integration (e.g., exercising with others), belonging, and the perceived legitimacy of prioritizing health within student role expectations. In other words, health-related practices carry symbolic meanings—discipline, attractiveness, productivity, sociability—that are unequally distributed and policed within peer cultures, making them socioculturally regulated practices rather than purely health-motivated actions. Framed as competencies, such practices depend not only on individual motivation or knowledge but also on whether peer contexts provide recognition, support, and norms that render these practices feasible and socially legitimate.

The university’s role as a social and cultural institution adds an additional layer of determination. Universities do not merely deliver education; they organize time, regulate space, concentrate peer interactions, and communicate implicit standards about performance, identity, and success. The institutional structuring of daily life—assessment cycles, workload intensification, residence arrangements, and campus mobility patterns—can create predictable “risk windows” for unhealthy routines (e.g., sleep curtailment during examinations, reduced activity during high workload periods) and normalize coping strategies that are socially endorsed within student communities. Environmental evidence supports this institutional framing: features such as walkability, active transportation infrastructure, and exposure to campus greenness are associated with health-relevant outcomes, including physical activity and mental restoration (Ding et al., 2024). Complementing this, a systematic review focusing on natural environments in university campuses reported consistently positive associations between campus greenery and students’ wellbeing, arguing that campus design can function as a population-level resource rather than relying solely on individualized interventions (Ribeiro et al., 2024). Taken together, these studies imply that the “university effect” on health lifestyles—and on wellbeing-related competencies—is not limited to individual stress or attitudes but includes institutional design, policy, governance, and cultural climate.

Despite a rapidly growing empirical literature, sociological evidence remains fragmented across health domains (e.g., alcohol, physical activity, mental wellbeing), disciplines (public health, psychology, urban studies, sociology), and levels of explanation (individual attitudes vs. peer networks vs. institutional arrangements). This fragmentation makes it difficult to specify which sociocultural determinants are most consistently associated with healthy lifestyle practices, how they interact (e.g., norms × resources; norms × stratification), and where universities can intervene most effectively—through curriculum, campus environment, student services, and institutional governance. Because the present manuscript is positioned as a sociological review, it prioritizes explanations that connect micro-level practices to meso-level institutional contexts and macro-level inequalities, consistent with contemporary directions in health lifestyles research (Mollborn et al., 2021). At the same time, aligning the review with the sustainable development competency agenda requires making explicit how these sociological mechanisms shape students’ capacities for self-regulation, wellbeing maintenance, and responsible participation within higher education.

Accordingly, this review synthesizes recent scholarship on social norms and healthy lifestyle practices among university students from a sociological perspective, with an explicit focus on the institutional conditions under which wellbeing-related competencies are formed, supported, or eroded. We examine how norms and normative climates shape patterns of health practice, how peer ecologies and transitions restructure those norms, and how universities—as institutions with distinctive organizational logics—enable or constrain health-supportive routines. To ensure transparency and reproducibility, the review is reported in accordance with PRISMA 2020 guidance (Page et al., 2021). By consolidating evidence across health practice domains and clarifying sociological mechanisms, the review aims to provide:

(i) An integrated account of sociocultural determinants (especially social norms) relevant to wellbeing-related competencies in university education,
(ii) An analytic framework linking peers, culture, and institutional context to student health lifestyles as competence-related outcomes, and
(iii) Implications for university-level strategies that move beyond individual behavior change toward structural, cultural, and governance levers.

This article contributes to sociology by theorizing universities as normative institutions that organize students’ wellbeing through temporal, digital, and moral regulation.

## 2 Materials and methods

### 2.1 Review design and data sources

This study employs a systematic review methodology to synthesize sociological research on the sociocultural and institutional determinants of healthy lifestyle practices among university students, with particular attention to how these determinants shape wellbeing-related capacities that are increasingly discussed as transferable competencies in higher education. A systematic approach was selected to ensure transparency, analytical rigor, and reproducibility, while allowing for the integration of diverse theoretical perspectives and empirical traditions within sociology. Unlike intervention-oriented reviews commonly found in public health research, the present review prioritizes sociological explanation, conceptual coherence, and the identification of social mechanisms shaping health-related practices in higher education contexts.

The review process was guided by established methodological principles for systematic reviews in the social sciences, emphasizing explicit selection criteria, structured synthesis, and reflexive interpretation of findings (Booth et al., 2016). Reporting followed the logic of the PRISMA 2020 framework, adapted for qualitative, quantitative, and mixed-methods sociological studies, where the aim is not statistical aggregation but theoretical integration and thematic synthesis (Page et al., 2021).

A comprehensive literature search was conducted across major international academic databases, including Scopus, Web of Science (Core Collection), Sociological Abstracts, and Google Scholar. These databases were selected to capture a broad range of sociological scholarship on student health, everyday practices, and institutional contexts, including literature that links university environments and norms to students’ self-regulatory and wellbeing-related capacities. The search strategy combined terms related to social norms, healthy lifestyle practices, culture, and higher education using Boolean operators and truncation to account for terminological variation. Searches were limited to publications in English and to the period from 2010 to 2025 in order to reflect recent theoretical developments and contemporary empirical evidence.

The literature search was conducted between January and March 2025. The search strategy combined controlled vocabulary and free-text keywords using Boolean operators (“AND,” “OR”) and truncation to capture terminological variation across sociology, higher education, and health research. The principal search string included combinations such as: (“healthy lifestyle” OR wellbeing OR “health behavior” OR “health practice”) AND (“student” OR “university” OR “higher education”) AND (“social norm” OR “institution”).

**Figure 1:**
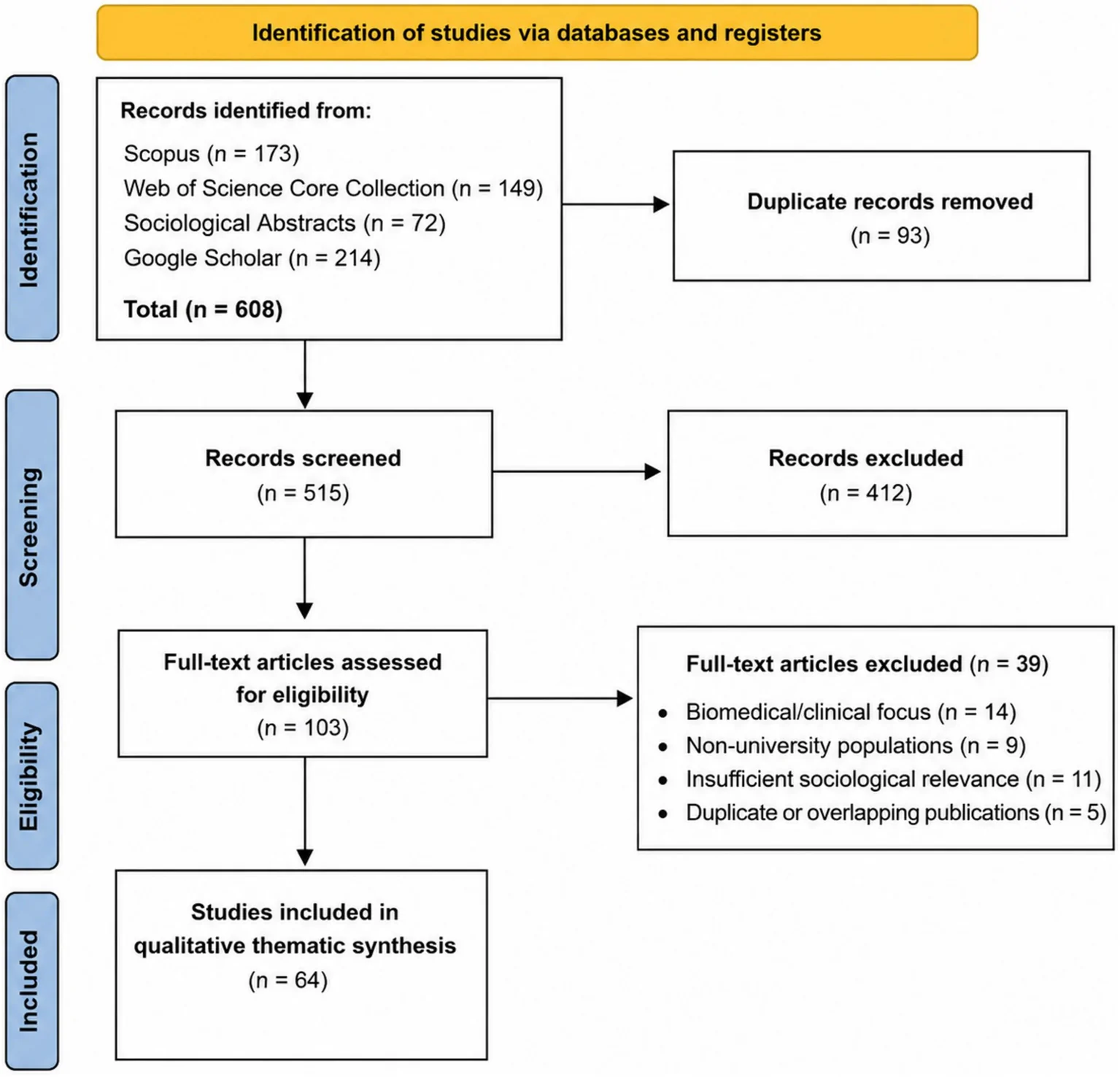
PRISMA flow diagram illustrating the study selection process.

The database search identified 608 records (Scopus, n = 173; Web of Science Core Collection, n = 149; Sociological Abstracts, n = 72; Google Scholar, n = 214). After removing 93 duplicate records, 515 titles and abstracts were screened. Following the exclusion of 412 records that did not meet the eligibility criteria, 103 full-text articles were assessed for eligibility. Of these, 39 publications were excluded after full-text review because they focused exclusively on biomedical or clinical perspectives, investigated non-university populations, lacked explicit sociological relevance, or represented duplicate or overlapping evidence. Ultimately, 64 studies met all eligibility criteria and were included in the final thematic synthesis. The study selection process is presented in Figure 1.

### 2.2 Selection and eligibility criteria

Eligibility criteria were applied to ensure the sociological relevance and analytical depth of the included studies. Only peer-reviewed journal articles and scholarly books or book chapters were considered. Studies were included if they focused on university or higher education students and explicitly engaged with sociocultural, institutional, or structural determinants of health-related practices, including the normative and organizational conditions under which such practices become routine, legitimate, and sustainable within student life. Research grounded exclusively in biomedical or clinical frameworks, intervention-only evaluations without sociological analysis, studies of non-student populations, and non-peer-reviewed materials were excluded.

The study selection process involved an initial screening of titles and abstracts, followed by full-text assessment of potentially relevant publications. Selection decisions were based on theoretical relevance, clarity of sociological framing, and the extent to which studies contributed to understanding healthy lifestyle practices as socially organized phenomena within university education. Throughout the screening process, inclusion criteria were applied iteratively to maintain consistency and analytical focus. The eligibility assessment was conducted in two sequential stages. First, titles and abstracts were screened according to the predefined inclusion criteria. Potentially eligible publications subsequently underwent full-text review. Reasons for exclusion during full-text assessment included studies focusing exclusively on biomedical or clinical interventions, non-university populations, insufficient sociological relevance, and duplicate or overlapping publications.

### 2.3 Data extraction, synthesis, and quality appraisal

Data extraction was conducted with attention to both conceptual and empirical dimensions of the selected literature. For each study, information was recorded on the theoretical framework employed, the institutional and cultural context of the research, methodological approach, and key findings related to sociocultural and institutional determinants of healthy lifestyle practices (and, where relevant, the implications for students’ capacities for self-regulation, wellbeing maintenance, and help-seeking). Rather than aggregating outcomes, the synthesis followed a thematic analytical strategy, enabling the identification of recurring concepts, explanatory patterns, and points of divergence across studies. This approach is particularly suited to systematic reviews in sociology, where heterogeneity of methods and contexts is expected and theoretically meaningful (Braun and Clarke, 2022).

Quality appraisal was carried out in a design-sensitive manner, recognizing the diversity of methodological approaches within sociological research. Studies were assessed in terms of theoretical coherence, methodological transparency, and analytical rigor, without applying rigid scoring systems.

Theoretical coherence was evaluated according to four predefined criteria:

(i) Consistency between the theoretical framework and research objectives;
(ii) Explicit conceptualization of key sociological constructs;
(iii) Logical correspondence between empirical findings and theoretical interpretation; and
(iv) Contribution to understanding institutional and sociocultural mechanisms influencing student wellbeing.

This design-sensitive appraisal approach was considered more appropriate than quantitative scoring because of the methodological diversity of the included studies. This flexible appraisal strategy supports interpretative synthesis while maintaining critical engagement with the reviewed literature (Gough et al., 2017).

Several limitations of the review should be acknowledged. Restricting the search to English-language publications may have excluded relevant studies published in other languages, and the focus on peer-reviewed literature may underrepresent emerging perspectives found in institutional or policy reports. Nevertheless, the systematic and transparent methodology provides a robust foundation for synthesizing sociological evidence and supports the validity of the thematic analysis presented in the following section.

The principal characteristics of the studies included in the final thematic synthesis are summarized in Table 1, providing an overview of their geographical distribution, methodological approaches, and thematic contribution to the review.

**Table 1:** Characteristics of the studies included in the final thematic synthesis.

References	Country/Region	Study design	Main focus	Contribution to the review
Exelmans and Van Den Bulck (2016)	Belgium	Cross-sectional study	Sleep behavior and media use	Digital habits and sleep-related practices among university students
Morrish (2019)	United Kingdom	Conceptual analysis	Neoliberal university culture	Institutional norms and academic pressure
Selwyn and Jandrić (2020)	International	Theoretical review	Digital higher education	Digital institutional environment and student wellbeing
Wunsch et al. (2021)	Germany	Longitudinal study	Stress and sleep	Academic workload and health-related behaviors
Li et al. (2022)	China	Cross-sectional study	Alcohol-related social norms	Peer influence on health practices
Brewster et al. (2022)	United States	Qualitative study	Academic culture	Institutional expectations and wellbeing
Varma et al. (2022)	International	Systematic review	Student mental health support	Institutional support mechanisms
Kim (2023)	South Korea	Survey study	Preventive health behavior	Institutional health promotion strategies
Hyseni Duraku et al. (2023)	Kosovo	Cross-sectional study	Help-seeking behavior	Institutional trust and access to support services
Gao et al. (2024)	China	Systematic review	Physical activity	Campus environment and physical activity promotion
Wang et al. (2024)	China	Meta-analysis	Physical activity	University-based health promotion initiatives
Jurado-Gonzalez et al. (2025)	Spain	Cross-sectional study	Healthy eating behaviors	Food environment and dietary practices among university students

## 3 Social norms, culture, and everyday practices: a sociological framework

The thematic synthesis identified three interrelated sociological dimensions through which universities shape healthy lifestyle practices among students. Together, these dimensions address the objective of the review by explaining how institutional normativity operates through cultural meanings, structural inequalities, and organizational environments to influence everyday health-related practices. This integrated framework provides the basis for the thematic presentation of the findings in the following sections.

Healthy lifestyle practices among university students are socially patterned forms of everyday activity shaped by normative expectations, cultural meanings, and institutional arrangements. Sociological research consistently demonstrates that behaviors related to physical activity, diet, sleep, stress management, and help-seeking are embedded in social relations and collective routines rather than determined solely by individual preferences or health knowledge. In higher education settings, dense peer networks, visibility of behavior, and structured academic rhythms intensify normative influence, making social norms a central mechanism not only in the organization of student health practices but also in the formation of wellbeing-related capacities that can be understood as competencies relevant to sustainable development.

Social norms regulate behavior by establishing shared expectations about what is typical and what is socially approved within a given group. Empirical research distinguishes between descriptive norms, which reflect perceptions of how others behave, and injunctive norms, which reflect perceptions of social approval or disapproval. Both forms of norms have been shown to shape health-related practices among university students, often independently of personal attitudes or health knowledge. From a competency-oriented perspective, these norms influence which practices students can sustain over time and integrate into their everyday academic participation.

Recent studies demonstrate that descriptive norms are particularly influential in contexts where behaviors are observable and socially salient. Evidence from student populations shows that perceived peer behavior predicts compliance with health-related practices, including preventive actions, even when controlling for individual risk perceptions and beliefs (Betsch et al., 2020; Jaffe et al., 2022). Similar mechanisms have been documented in research on alcohol use, where students’ overestimation of peers’ consumption contributes to the normalization of risky patterns and the persistence of unhealthy routines (Borsari and Carey, 2003). These findings indicate that norms function as collective reference points through which students calibrate acceptable conduct, thereby shaping not only immediate behavior but also longer-term dispositions related to self-regulation and wellbeing maintenance.

Normative influence also extends to health-promoting practices. Meta-analytic evidence indicates that social support and peer-related normative climates are positively associated with physical activity among university students across diverse cultural contexts (Wang et al., 2024). Systematic review evidence further shows that social capital, including trust, cohesion, and network participation, facilitates engagement in physical activity through mechanisms such as role modeling and shared expectations (Gao et al., 2024). Together, these studies support a sociological interpretation of norms as relational processes embedded in social networks rather than as isolated cognitive perceptions, highlighting their role in enabling or constraining the development of sustainable health-related routines.

Contemporary theoretical work conceptualizes norm perception as a multi-layered process shaped by interpersonal interactions, institutional signals, and organizational routines rather than as a static individual belief. Organizational research demonstrates that perceptions of collective norms emerge through dynamic interpretive processes influenced by group expectations and formal structures (Dannals and Li, 2024). This perspective is particularly relevant to university settings, where normative expectations are not only communicated among peers but also reinforced through institutional messaging, assessment systems, and structured academic rhythms. As a result, universities contribute to the stabilization of particular patterns of practice that may support—or undermine—the acquisition of wellbeing-related competencies.

**Figure 2:**
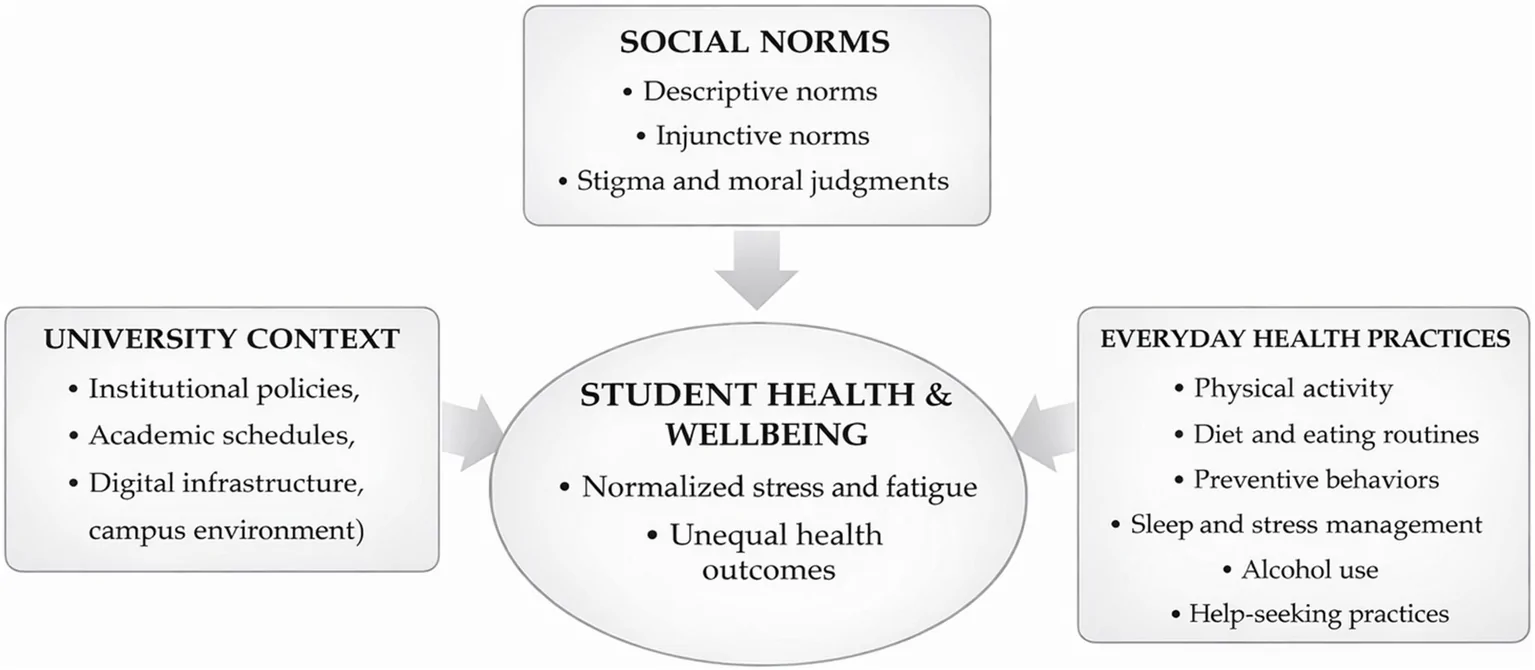
Conceptual framework illustrating the relationships between institutional context, social norms, everyday health practices, and student health and wellbeing in higher education.

The relationships between institutional contexts, social norms, and everyday health-related practices discussed in this section are summarized in Figure 2.

### 3.1 Culture, meaning, and health practices as social routines

Health-related practices acquire meaning within cultural frameworks that define what counts as legitimate, respectable, or desirable behavior in student life. Cultural norms shape the moral evaluation of practices such as sleep, productivity, exercise, and self-care, often linking them to identities of success, discipline, or resilience. In many university contexts, chronic stress, irregular sleep, and high workload tolerance become normalized as markers of academic commitment, while practices oriented toward recovery or help-seeking may be culturally marginalized. These cultural valuations are critical because they determine which forms of self-care are recognized as compatible with being a “good student,” and which are implicitly discouraged.

Practice-oriented sociology conceptualizes health-related behaviors as routinized configurations of meanings, competences, and material arrangements. From this perspective, practices persist because they are supported by infrastructures, skills, and shared understandings rather than because individuals repeatedly choose them anew. Review-level scholarship demonstrates that practices form interconnected bundles, such that changes in one domain (e.g., academic workload) can cascade across others, including sleep, diet, and physical activity (Klitkou et al., 2022). This insight helps explain why fragmented interventions targeting single behaviors often fail to produce sustained change: they do not alter the broader configurations through which competencies are enacted in everyday life.

Empirical research among university students supports this practice-based interpretation. Studies show that lifestyle patterns are strongly associated with relational contexts, including family dynamics and perceived social support, even during emerging adulthood (Domingues et al., 2024). Additional sociological analyses demonstrate that access to time, space, and socially legitimate opportunities conditions participation in health-promoting practices, reinforcing the role of structural and cultural resources in shaping student lifestyles (Spotswood and Gurrieri, 2023). From a competency perspective, these findings suggest that students’ ability to maintain healthy routines depends on the availability of supportive practice environments rather than on individual motivation alone.

Recent theoretical contributions further integrate practice theory with health sociology by framing health as a biosocial process constituted through everyday practices that simultaneously shape bodies and social relations (Shove et al., 2024). This approach provides a coherent basis for analyzing multiple lifestyle domains within a single framework, without reducing health to either biological outcomes or subjective meanings, and aligns with understandings of wellbeing as a competence that develops through repeated participation in socially organized practices.

### 3.2 Health lifestyles and patterned inequalities in student populations

Health lifestyle theory offers a unifying framework that links social norms, cultural practices, and structural conditions into durable patterns of behavior. Contemporary developments in this theory highlight the influence of digitalization, institutional change, and global health risks on everyday routines, all of which are highly relevant to current university populations (Cockerham, 2023). Within this framework, student health practices reflect the interaction of life chances—including socioeconomic position and institutional context—with socially shaped dispositions and choices.

Empirical evidence consistently shows that health-related practices among students are stratified by gender, socioeconomic background, housing conditions, and study arrangements. These patterned differences indicate that healthy lifestyle practices, and the competencies associated with sustaining them, are unevenly distributed across student groups. Such inequalities reinforce the need for a sociological analysis that accounts for structural differentiation, institutional context, and unequal access to health-supportive resources rather than attributing health outcomes solely to individual responsibility or resilience.

The reviewed literature also suggests that socioeconomic disparities influence students’ capacity to adopt and sustain healthy lifestyle practices through multiple interconnected mechanisms. Financial constraints may limit access to nutritious food, recreational facilities, or organized physical activities, while employment during study often reduces the time available for sleep, exercise, and recovery. Differences in housing quality, commuting demands, and access to campus resources further contribute to unequal opportunities for maintaining healthy routines. These findings indicate that health-related practices are embedded within broader social and institutional structures, reinforcing the argument that student wellbeing cannot be understood solely as an outcome of individual motivation or personal responsibility (Hyseni Duraku et al., 2024; Ding et al., 2024; Dooris et al., 2021).

### 3.3 Universities as normative and infrastructural environments

Universities function as social institutions that actively structure health-related practices by organizing time, space, and social interaction. Academic calendars, assessment systems, housing arrangements, and campus infrastructures create conditions that enable or constrain daily routines. Systematic review evidence demonstrates that built and natural campus environments—such as walkability, transport infrastructure, and access to green spaces—are associated with physical activity and mental well-being among students (Ding et al., 2024; Ribeiro et al., 2024). These findings underscore the importance of material and spatial arrangements in shaping everyday practices through which wellbeing-related capacities are enacted.

At the organizational level, institutional commitments to health and wellbeing vary substantially across universities. Analyses of health-promoting university strategies show that when wellbeing is integrated into governance and policy, it can influence campus norms and routines; however, many initiatives remain fragmented and unevenly implemented (Dooris et al., 2021; Pani et al., 2025). Sociological research on the hidden curriculum further demonstrates that informal expectations—such as the normalization of overwork—can undermine formal health promotion efforts by reproducing contradictory norms within academic culture (Rossouw and Frick, 2023).

Taken together, sociological evidence indicates that healthy lifestyle practices among university students emerge from the interaction of social norms, cultural meanings, practice configurations, and institutional structures. This integrated framework provides a coherent basis for analyzing empirical findings across diverse health domains while maintaining a consistent sociological focus on norms, culture, and inequality. At the same time, it clarifies how universities shape the conditions under which wellbeing-related competencies—central to sustainable development in higher education—are formed, sustained, or constrained.

## 4 Thematic findings: social norms and everyday health practices in university life

Building on the sociological framework presented in the previous section, the following thematic findings illustrate how institutional normativity is manifested across different domains of student health practices. To address the objective of this review, the thematic findings are organized according to the principal domains through which institutional normativity shapes healthy lifestyle practices among university students. Sociological research consistently demonstrates that behaviors related to physical activity, diet, sleep, stress management, and help-seeking are embedded in social relations and collective routines rather than determined solely by individual preferences or health knowledge. In higher education settings, dense peer networks, visibility of behavior, and structured academic rhythms intensify normative influence, making social norms a central mechanism not only in the organization of student health practices but also in the formation of wellbeing-related.

### 4.1 Normative regulation of visible health practices in student communities (physical activity, prevention, and diet)

In university settings, the health practices that are most *publicly observable*—such as exercising on campus, eating in shared spaces, and complying with prevention routines—are especially prone to normative regulation. This is because students’ everyday life is organized around dense peer networks, repeated encounters, and semi-public arenas (campus facilities, dormitories, cafeterias, student groups, and digitally mediated peer spaces). In such contexts, health-related action is not only a matter of personal preference or knowledge, but also a form of socially monitored conduct that signals belonging, competence, and “fitting in” with the prevailing student culture. Empirically, this is one reason why perceived peer expectations and relational resources (e.g., support and network cohesion) repeatedly emerge as correlates of students’ lifestyle practices across countries and institutional types (Gao et al., 2024; Wang et al., 2024). From a competency-oriented perspective, these visible practices are key sites where wellbeing-related capacities are enacted, recognized, or discouraged within everyday academic participation.

A core sociological mechanism here is the distinction between descriptive norms (beliefs about what peers typically do) and injunctive norms (beliefs about what peers approve/disapprove). In campus life, these norms are continuously produced and reinforced through visibility: who goes to the gym, what is considered “normal” food in the cafeteria, or what prevention behaviors are practiced during outbreaks. Norms become actionable because students often rely on peer conduct as a heuristic for what is appropriate in ambiguous situations, particularly under conditions of uncertainty and competing demands such as academic workload, social obligations, and time scarcity. At the same time, normative influence is increasingly shaped by hybrid social spaces in which offline observation intersects with online signaling—such as posting workouts, food routines, or “wellness” identity cues—thereby extending peer comparison and reputational stakes beyond face-to-face interaction (Patwardhan et al., 2024). These processes shape not only immediate behavior but also students’ perceived ability to sustain health-related routines over time.

Physical activity represents one of the clearest examples of a visible health practice governed by peer climates. A recent meta-analysis among college and university students shows that social support is reliably associated with physical activity, indicating that students’ movement behaviors are not simply individual “choices” but relationally scaffolded practices involving encouragement, co-participation, and normative approval (Wang et al., 2024). Complementary systematic review evidence focusing on social capital demonstrates that network strength, cohesion, and participation are linked with higher levels of physical activity and explicitly interprets these associations through social mechanisms such as role modeling and norm formation (Gao et al., 2024). From a sociological standpoint, these findings suggest that “being active” functions as a socially supported routine in some student communities, while in others the absence of supportive norms and collective arrangements—such as shared time, safe routes, or inclusive group cultures—renders inactivity more socially sustainable than activity. As a result, opportunities to develop sustainable movement-related competencies are unevenly distributed across institutional and social contexts.

The campus environment further amplifies normative regulation by shaping which practices are easy to display, repeat, and integrate into daily routines. A systematic review of built and natural campus environments links walkability, intersections, and infrastructure for active transport to student physical activity, and associates campus greenness and blue space with wellbeing outcomes (Ding et al., 2024). In practical terms, when campus design makes walking and active transport routine, the descriptive norm shifts: movement becomes a default and socially expected baseline rather than a specialized “health project.” Conversely, when the campus setting restricts visible opportunities for activity—or renders them socially segmented—physical activity may become normatively polarized, highly salient in certain groups while remaining marginal in others.

Preventive behaviors, such as masking, hygiene routines, and physical distancing during outbreaks, illustrate how rapidly norms can reorganize visible practices under conditions of institutional and peer surveillance. Research on preventive behavior consistently shows that social and cognitive factors jointly shape compliance, with norms and social beliefs functioning as central pathways. For example, a study of Korean college students demonstrates how social beliefs relate to COVID-19 precautionary behaviors through health-belief mechanisms, indicating that compliance is embedded in shared understandings of governance and collective responsibility rather than reducible to individual risk attitudes alone (Kim, 2023). Large-scale research among higher education students similarly documents that adherence to sanitary behaviors varies systematically with risk perception and related psychosocial factors, reinforcing that compliance is patterned and responsive to perceived social expectations (Dekeyser et al., 2023). At a broader level, evidence informed by the theory of planned behavior shows that subjective norms are independently associated with multiple preventive actions in pandemic contexts (Aschwanden et al., 2021). Together, these findings justify treating prevention not as a purely biomedical behavior, but as a normatively regulated campus practice—one that is sustained when institutional and peer expectations converge and weakened when signals are fragmented or contested.

Dietary practices on campus are equally visible and norm-sensitive, but they are governed by an additional layer: the university food environment. A systematic review in *Frontiers in Nutrition* synthesizes evidence that the taste, price, and availability of campus food options shape student choices, and also highlights a recurrent perception problem—students may interpret unhealthy options as “healthy,” suggesting that the meaning of “healthy eating” is socially negotiated within campus routines rather than simply known and applied (Li et al., 2022). More recent mixed-methods evidence illustrates how academic stress, time constraints, and peer pressure/negative social norms can contribute to poorer diet quality and irregular eating patterns—especially for students living independently, where peer routines often substitute for family-regulated food structures (Jurado-Gonzalez et al., 2025). A sociological implication is that campus eating is not merely an individual nutrition issue but a public practice embedded in social timing, shared spaces, and reputational dynamics. Under these conditions, norms can normalize convenience foods, energy drinks, or skipping meals as part of “student life,” while simultaneously valorizing selective “healthy” performances (e.g., fitness diets) within particular subgroups.

Finally, contemporary theory emphasizes that normative influence is not a single-level phenomenon but is produced across interpersonal interaction, organizational settings, and digitally networked communication. This multilevel understanding is essential for analyzing visible health practices in universities because it explains why peer norms may be amplified or counteracted by institutional messaging, assessment regimes, and online community dynamics rather than operating as isolated perceptions within individuals (Dannals and Li, 2024). Within a PRISMA-reported review, this framework provides a coherent analytic lens for synthesizing heterogeneous evidence—ranging from qualitative accounts of campus routines to cross-sectional surveys and systematic reviews—into a unified sociological explanation. Visible health practices become “sticky” when they are routinized in shared spaces, supported by social capital and infrastructure, and reinforced through repeated normative cues, both offline and online. In this way, universities shape not only what students do, but what forms of health-related competence they are realistically able to sustain during their studies.

Whereas the previous section examined health practices that are publicly visible and strongly influenced by peer norms, the following section considers less visible but equally pervasive practices related to stress, fatigue, and sleep that are primarily shaped by institutional rhythms and academic expectations.

### 4.2 Normalization of stress, sleep deprivation, and academic overload

In contemporary higher education, stress, chronic fatigue, and irregular sleep are widely normalized as routine features of student life, with direct implications for students’ capacity to sustain learning, wellbeing, and participation over time. Sociological research increasingly interprets these patterns not as individual maladaptation, but as the outcome of shared expectations, institutional rhythms, and cultural narratives that frame endurance and constant productivity as markers of academic legitimacy. Empirical studies across regions consistently report high levels of perceived stress and sleep problems among university students, indicating that these experiences are structurally patterned rather than episodic or exceptional (Beiter et al., 2015; Reddy et al., 2015). Importantly, students often interpret stress and exhaustion as unavoidable or even necessary for academic success, which contributes to their social normalization within university cultures.

A growing body of evidence demonstrates that academic overload is a central driver of both stress and sleep deprivation. Quantitative studies show strong associations between perceived academic demands, time pressure, and psychological distress among undergraduates and graduate students, even after controlling for individual coping styles (López-Castro et al., 2021; Pascoe et al., 2020). Longitudinal data further suggest that stress levels peak during periods of assessment density, reinforcing the role of institutional scheduling in shaping collective experiences of strain (Wunsch et al., 2021). From a sociological perspective, these findings highlight how stress becomes routinized through shared academic calendars and synchronized deadlines, producing a collective rhythm in which exhaustion is temporally concentrated and socially anticipated.

Sleep deprivation is closely intertwined with these dynamics and has become one of the most pervasive yet normalized health disruptions in student populations. Large-scale surveys consistently report insufficient sleep duration and poor sleep quality among university students, with prevalence rates that exceed those observed in age-matched non-student populations (Hershner and Chervin, 2014). Crucially, qualitative research indicates that students often perceive sleep loss as a rational and socially sanctioned trade-off for academic performance, particularly during examination periods (Foulkes et al., 2019; Li et al., 2022). This framing reflects a cultural logic in which sleep is treated as flexible and expendable, rather than as a non-negotiable biological requirement.

The digitalization of university life has further intensified the normalization of stress and sleep deprivation. Empirical studies link extensive use of digital learning platforms, constant connectivity, and late-night engagement with academic and social media content to disrupted sleep patterns and heightened cognitive arousal (Al-Shahrani, 2025; Garett et al., 2018; Scott and Woods, 2019). Social media and messaging applications function not only as tools for communication but also as normative infrastructures that reinforce expectations of continuous availability and rapid response. Evidence from cross-sectional and longitudinal studies shows that perceived pressure to remain digitally engaged—both academically and socially—is associated with later bedtimes, reduced sleep duration, and increased stress among students (Exelmans and Van Den Bulck, 2016; Lemola et al., 2015). These patterns suggest that digital environments amplify existing academic pressures by extending them temporally into the night.

Cultural narratives of productivity play a critical role in legitimizing these practices. Sociological analyses describe how “busyness” and overcommitment are frequently valorized within higher education as indicators of ambition, resilience, and merit, while rest and recovery are framed as secondary or indulgent (Chemagosi, 2024; Crawford, 2024). Survey research among students supports this interpretation, showing that individuals who endorse strong productivity norms report higher stress and are more likely to sacrifice sleep for academic tasks, even when aware of negative health consequences (Li et al., 2025). Such findings underscore that stress and sleep deprivation persist not because students lack information, but because cultural meanings attached to success encourage self-exploitation within accepted boundaries.

Importantly, the normalization of stress and fatigue is not evenly distributed across student populations. Studies indicate systematic differences by gender, socioeconomic background, and field of study, with students from less advantaged backgrounds and highly competitive programs reporting greater exposure to chronic stressors and fewer resources for recovery (De Los De Los Reyes et al., 2019; Elmer et al., 2020). These inequalities suggest that normalized overload functions as a mechanism through which broader social stratification is reproduced within higher education, reinforcing the need for a sociological lens that accounts for structural position rather than attributing outcomes to individual resilience. The reviewed evidence indicates that stress and sleep deprivation are sustained through recurring institutional and cultural mechanisms rather than isolated individual choices. Table 2 synthesizes the key sociocultural determinants identified in the literature, linking structural conditions to normative framings and observable health outcomes in university settings.

**Table 2:** Sociocultural determinants of stress and sleep deprivation among university students.

Determinant	Normative framing	Observable outcomes	Sociological interpretation	Key references
Academic workload	Productivity norm	Chronic stress	Normalization of overload	Wunsch et al. (2021)
Digital connectivity	Availability norm	Late bedtimes	Digital norm enforcement	Exelmans and Van Den Bulck (2016)
Assessment schedules	Performance norm	Sleep deprivation	Institutional rhythm	Reddy et al. (2015)

Thus, the reviewed evidence supports a coherent sociological interpretation of stress, sleep deprivation, and academic overload as collectively organized and culturally sanctioned aspects of university life. These practices are stabilized through institutional schedules, digital infrastructures, and normative ideals of productivity that render exhaustion both predictable and socially intelligible. Rather than isolated health behaviors, stress and sleep loss emerge as embedded components of everyday student routines, shaping the limits of students’ self-regulatory and wellbeing-related competencies and sustained through shared expectations and repeated interaction within academic environments. Beyond everyday routines such as sleep and workload management, institutional normativity also shapes practices that involve moral evaluation and social stigma, particularly alcohol use and mental health help-seeking.

### 4.3 Stigma, moral judgments, and help-seeking practices in university settings

Health-related practices that involve moral evaluation—most notably alcohol use and mental health help-seeking—occupy a distinctive normative position within university life. Unlike physical activity or diet, these practices are closely bound to judgments about responsibility, self-control, and social legitimacy. Sociological research shows that students navigate these domains by balancing competing norms: on the one hand, expectations of sociability and emotional resilience; on the other, norms of self-regulation, responsibility, and academic competence (Ishita Vyas et al., 2026). Empirical evidence indicates that these moralized expectations shape not only behavior but also students’ willingness to disclose difficulties and engage with institutional support systems, thereby influencing the development and use of wellbeing-related competencies (Sanchez et al., 2022).

Alcohol use among university students is best understood as a normatively regulated social practice rather than a purely individual risk behavior. Large-scale surveys and systematic syntheses demonstrate that drinking patterns are strongly associated with perceived peer norms and social contexts in which alcohol consumption functions as a mechanism of belonging and identity formation. A recent systematic review of university drinking cultures highlights that alcohol is often embedded in rituals of transition, celebration, and peer bonding, rendering abstention or moderation socially visible and potentially stigmatized (Newman et al., 2017; Paschall et al., 2009). Quantitative studies further show that perceived descriptive norms—beliefs about how much peers drink—are among the strongest predictors of individual consumption, even when controlling for personality traits and stress levels (Brunelle and Hopley, 2017; Sun et al., 2018). These findings support a sociological interpretation in which alcohol use is stabilized by shared expectations about what constitutes “normal student behavior,” rather than by individual preference alone.

At the same time, moral judgments surrounding alcohol are highly ambivalent. While heavy drinking may be normalized in some social settings, it is simultaneously subject to institutional and public health discourses that frame it as irresponsible or pathological. This tension is reflected in qualitative studies showing that students engage in moral boundary work, distinguishing between “acceptable” and “problematic” drinking based on context, intent, and social visibility rather than on health criteria alone (Dorn, 2023; Hebden et al., 2015). Such moral differentiation allows continued participation in drinking cultures while distancing individuals from stigmatized identities associated with addiction or loss of control.

Mental health help-seeking is shaped by parallel normative mechanisms but is more directly constrained by stigma and institutional trust. Despite rising prevalence of stress, anxiety, and depressive symptoms among university students, utilization of formal mental health services remains uneven. Systematic review evidence indicates that perceived stigma—both public and self-stigma—constitutes a persistent barrier to help-seeking across higher education contexts (Cheng et al., 2015; Mitchell et al., 2017). Importantly, stigma operates not only as fear of negative judgment but also as an internalized moral narrative in which psychological distress is interpreted as personal weakness or insufficient resilience, thereby constraining students’ capacity to mobilize available support.

Recent empirical studies deepen this understanding by demonstrating that students’ help-seeking intentions are shaped by perceived peer attitudes and cultural norms within academic environments. Multi-country survey evidence shows that students who believe their peers view counseling positively are significantly more likely to express willingness to seek professional help, independent of symptom severity (Ebert et al., 2019; Lally et al., 2013; Lui et al., 2024). These findings underscore that help-seeking is a socially situated practice governed by injunctive norms concerning which forms of vulnerability are legitimate within student culture.

Institutional trust emerges as a crucial mediator between stigma and help-seeking behavior. Research in higher education sociology shows that students’ confidence in the confidentiality, accessibility, and responsiveness of university support services strongly influences whether perceived need is translated into action. Mixed-methods evidence from European universities indicates that mistrust in institutional procedures—particularly concerns about academic consequences or lack of anonymity—significantly reduces help-seeking, even among students with high mental health literacy (Amozgar et al., 2024; Auerbach et al., 2016). Conversely, institutions that integrate wellbeing into governance and communicate supportive norms at multiple levels tend to normalize help-seeking as an acceptable and competent response to distress.

The intersection between alcohol norms and mental health stigma further illustrates the moral complexity of student health practices. Studies indicate that alcohol consumption is frequently used as an informal coping strategy for stress and emotional difficulties, particularly in contexts where help-seeking is stigmatized or perceived as inaccessible (Pedrelli et al., 2024; Walker and Stephens, 2014). This substitution effect reinforces existing drinking norms while delaying engagement with professional support. Sociologically, such patterns reflect the alignment of coping behaviors with socially sanctioned practices, even when these practices undermine long-term wellbeing.

Importantly, stigma and moral judgment do not affect all students equally. Research documents systematic differences by gender, socioeconomic status, and cultural background, with marginalized groups often experiencing higher stigma and lower institutional trust (Han and Pong, 2015; Mendoza et al., 2015). These inequalities indicate that normative regulation of help-seeking operates as a mechanism of social stratification within higher education, shaping unequal access to support and uneven development of wellbeing-related competencies.

Overall, alcohol use and help-seeking practices among university students are shaped by dense moral economies in which norms of sociability, responsibility, and resilience intersect. These practices are sustained not only by peer cultures but also by institutional signals that define which behaviors are acceptable, which vulnerabilities are legitimate, and which forms of support can be safely accessed. From a sociological perspective, stigma and moral judgment therefore function as central organizing forces of student health practices, with direct implications for students’ capacity to sustain self-regulation and wellbeing within university education. Taken together, these thematic findings demonstrate that student health practices cannot be understood independently of the institutional environments in which they occur.

## 5 The university as a normative environment

Universities constitute complex normative environments in which health-related practices are shaped through institutional organization, cultural expectations, and material–digital infrastructures. From a sociological perspective, higher education institutions do not merely provide a backdrop for student lifestyles but actively structure what forms of conduct are considered legitimate, expected, or necessary. Health-related behaviors therefore emerge as patterned responses to institutional norms embedded in academic routines, governance arrangements, and symbolic constructions of success, rather than as isolated individual choices. Understanding the university as a normative environment enables a shift from behavior-focused explanations toward analyses of how institutional systems produce, stabilize, and legitimize everyday health practices among students (Brewster et al., 2022).

These institutional mechanisms operate in combination, reinforcing health-related norms through governance, infrastructures, and organizational cultures. Table 3 synthesizes the key pathways through which universities function as normative environments shaping student health practices.

**Table 3:** Universities as normative environments shaping student health practices.

Institutional dimension	Normative mechanism	Health-related practices affected	Sociological implication	Key references
Academic governance	Performance and productivity norms	Stress tolerance, sleep curtailment	Moralization of overload	Brewster et al. (2022); Morrish (2019)
Assessment architecture	Temporal concentration of demands	Chronic fatigue, recovery avoidance	Institutional rhythmization	Kainat Rashid et al. (2025)
Digital infrastructure	Availability and responsiveness norms	Late-night study, screen overuse	Extension of academic time	Selwyn and Jandrić (2020)
Campus spatial design	Visibility and accessibility norms	Physical activity, social interaction	Infrastructural regulation	Aghabozorgi et al. (2024)
Student support governance	Legitimacy and trust norms	Help-seeking vs. concealment	Institutional trust mediation	Hyseni Duraku et al. (2023); Varma et al. (2022)

### 5.1 Institutional cultures, academic rhythms, and the moral regulation of student life

A central mechanism through which universities shape student health practices lies in institutional cultures that morally regulate academic engagement. Contemporary sociological research highlights that higher education increasingly operates within a moral economy that valorizes productivity, endurance, and self-responsibility. Within this framework, stress, fatigue, and sustained pressure are often interpreted as indicators of commitment and academic seriousness rather than as signals of institutional strain. Qualitative studies conducted across European and Anglo-American universities demonstrate that students internalize expectations of constant performance, frequently describing overload and exhaustion as “normal” or “unavoidable” aspects of legitimate student life (Brewster et al., 2022; Morrish, 2019).

These cultural norms are reinforced through institutional rhythms that structure time and attention. Academic calendars characterized by clustered assessments, cumulative deadlines, and limited recovery periods create synchronized cycles of high stress that are collectively anticipated and socially normalized. Empirical research shows that such temporal concentration of academic demands is systematically associated with elevated psychological distress and sleep disruption among students, independent of individual coping strategies (Alhamed, 2023; Kainat Rashid et al., 2025). From a sociological standpoint, these findings indicate that stress is not an incidental by-product of study but a routinized outcome of organizational design.

Institutional discourses surrounding excellence, employability, and competitiveness further contribute to the moralization of health-related practices. Studies analyzing university policy documents and student narratives reveal that wellbeing is frequently framed as an individual responsibility—something to be “managed” alongside academic obligations—rather than as a collective or structural concern. This framing legitimizes self-regulation strategies that prioritize performance over recovery and discourages practices perceived as incompatible with productivity, such as rest, disengagement, or help-seeking (Enwefa et al., 2020; Morrish, 2019). As a result, health-compromising routines become culturally intelligible and institutionally tolerated.

Importantly, these normative pressures are unevenly distributed across student populations. Sociological analyses consistently demonstrate that students from lower socioeconomic backgrounds, those engaged in paid work alongside study, and those enrolled in highly competitive programs experience intensified exposure to institutional stressors and reduced access to buffering resources (Jury et al., 2017; Lutfiu and Lutfiu Hoxha, 2024; Posselt, 2021). Such inequalities underscore the role of universities as stratifying institutions that shape differentiated health trajectories through normative expectations embedded in academic structures.

### 5.2 Universities as infrastructural and governance systems shaping health practices

Beyond cultural expectations, universities function as normative environments through material, digital, and governance infrastructures that condition everyday practices. Campus design, spatial accessibility, and availability of facilities shape how students move, socialize, rest, and engage with health-related activities. A growing body of interdisciplinary research demonstrates that campus environments with accessible green spaces, walkable layouts, and integrated social areas are associated with improved mental wellbeing and reduced stress among students (Aghabozorgi et al., 2024; Bai et al., 2024). These effects are sociologically significant because they operate through routine exposure and habitual use rather than deliberate health choices.

Digital infrastructures represent an increasingly influential normative layer. Learning management systems, institutional email, and online assessment platforms extend academic demands beyond physical campus boundaries, contributing to an “always-on” culture of availability. Empirical studies link institutional digital expectations to prolonged screen time, disrupted sleep patterns, and blurred boundaries between academic and personal life (Büchi et al., 2019; Selwyn and Jandrić, 2020). From a normative perspective, digital connectivity becomes framed as academic responsibility, reinforcing health-compromising practices while remaining institutionally sanctioned.

Governance arrangements further mediate how health practices are normalized within universities. While many institutions have adopted wellbeing strategies or student support services, systematic reviews reveal that such initiatives are often weakly embedded within core governance structures and insufficiently aligned with teaching, assessment, and performance management systems (Bannigan et al., 2025; Tafireyi and Grace, 2024). This disjunction generates contradictory normative signals: universities promote wellbeing rhetorically while maintaining organizational practices that reproduce stress and overload.

Institutional trust emerges as a key mechanism linking governance structures to student behavior. Research shows that students’ willingness to engage with formal support services depends on perceptions of confidentiality, legitimacy, and alignment between institutional values and everyday practices. Where trust is low, students are more likely to rely on informal coping strategies and peer support, reinforcing norms of self-management and concealment (Hyseni Duraku et al., 2023; Varma et al., 2022). These dynamics illustrate how governance failures can indirectly shape health practices by influencing which forms of support are considered safe or acceptable.

Overall, these findings position the university as a multi-layered normative system in which cultural expectations, infrastructural arrangements, and governance practices converge to organize student health-related behavior. Rather than functioning as neutral settings, universities actively produce and stabilize patterns of practice that normalize stress, regulate visibility of vulnerability, and contribute to unequal health outcomes. This institutional perspective provides a critical bridge between the sociological frameworks discussed earlier and the integrative discussion that follows, emphasizing that sustainable improvements in student wellbeing require systemic, not merely individual-level, change.

## 6 Discussion and implications: why a sociological review of healthy lifestyle culture in universities matters

Building on the thematic findings and the institutional analysis presented above, this discussion interprets healthy lifestyle culture within broader sociological debates on normativity, governance, and social reproduction in higher education. This review set out to synthesize evidence on how healthy lifestyle culture among university students is socially produced and maintained through norms, institutional routines, and culturally meaningful definitions of “good” student conduct. Across the included literature, a consistent pattern emerges: student health practices (sleep, stress management, help-seeking, and everyday self-care) are not merely private choices but are organized through normative expectations and institutional infrastructures, thereby shaping students’ capacity to sustain self-regulation, wellbeing, and academic participation over time. The value of a sociological review lies precisely in making visible how universities function as environments that normalize particular rhythms of life—especially under conditions of intensified academic performance demands and digitally mediated student routines.

A first key finding is that “academic stress” cannot be treated as a purely individual psychological response when it is systematically linked to institutional pressures and their temporal organization. Empirical work in student populations shows robust associations between higher academic stress and poorer mental wellbeing, particularly in pandemic-accelerated academic settings where uncertainty and workload intensification were salient. For example, Barbayannis et al. (2022) report significant correlations between academic stress (measured via PAS) and lower mental wellbeing (SWEMWBS), and they highlight that stress responses vary across student subgroups and study stages, indicating structured vulnerability rather than random individual difference (Barbayannis et al., 2022). Complementing this, Yang et al. (2021) used a three-wave lagged design and found that academic workload had negative effects on student health through perceived stress, demonstrating how workload—an institutional output—translates into embodied outcomes via stress processes (Yang et al., 2021). Read sociologically, these studies support an interpretation where universities generate patterned exposures (workload density, assessment clustering, constant evaluation), and students adapt through normalization of overload rather than through deliberate “unhealthy choices.”

A second theme concerns the normalization of sleep deprivation and “always-on” digital availability as culturally regulated adaptations to university life. Digital connectivity is not simply a technological feature; it becomes a normative infrastructure that reorganizes time boundaries, study rhythms, and peer expectations of responsiveness. Large-scale longitudinal evidence illustrates how digitally mediated routines link to sleep deprivation and performance. Lin and Zhou (2022) analyzed a two-year longitudinal dataset of 6,093 college students and showed that bedtime smartphone use is associated with poorer academic performance through the mediators of sleep deprivation and nomophobia, providing a clear pathway from digital behavior embedded in daily routines to sleep loss and academic outcomes (Lin and Zhou, 2022). This is important for sociological interpretation because it moves beyond “screen time” as an individual habit and toward digitally enforced availability norms: when study and social belonging are mediated by devices, disengagement is socially costly, and sleep becomes the adjustable resource. Related evidence at the institutional level shows that digital overload and excessive platform use in university life can undermine wellbeing and shape coping practices; recent work in SAGE Open conceptualizes digital overload as a structured condition of contemporary student engagement rather than an idiosyncratic problem (Tafesse et al., 2024). The implication for a sociological understanding of healthy lifestyle culture is that sleep deprivation becomes normalized not only because students “choose” it, but because digitally mediated academic environments redefine the limits of sustainable self-regulation.

A third integrative finding concerns the institutional contradiction between wellbeing discourse and performance governance. Universities increasingly communicate a commitment to student wellbeing, yet many of the same institutions retain assessment architectures and productivity expectations that intensify overload. The sociological consequence is not just stress but moralization: students learn that wellbeing is a personal responsibility to be managed without disrupting performance. Evidence-based policy syntheses underscore that higher education institutions are dealing simultaneously with mental health, substance use, and wellbeing challenges at scale, requiring system-level responses rather than optional supports. A prominent example is the National Academies report Mental Health, Substance Use, and Wellbeing in Higher Education: Supporting the Whole Student (2021), which frames student wellbeing as an institutional responsibility and emphasizes whole-system approaches (policy, services, learning environments), not only individual interventions (Committee on Mental Health, Substance Use, and Wellbeing in STEMM Undergraduate and Graduate Education et al., 2021). Even when student-facing services exist, the cultural legitimacy of using them depends on whether institutional signals (through workload policy, flexibility, and staff practices) align with wellbeing messaging. Where misalignment persists, help-seeking can remain stigmatized or morally framed as weakness, especially in competitive environments.

A fourth theme is that stigma and help-seeking are socially patterned by cultural norms and institutional trust, not reducible to “lack of awareness.” Recent empirical research in university populations shows that stigma and fear of negative judgment strongly shape disclosure and help-seeking, particularly among students navigating cross-cultural academic contexts. Cogan et al. (2024), focusing on Asian international students in higher education, document how taboo, stigma, and fear of negative consequences constrain disclosure and engagement with support, highlighting the importance of social context and perceived institutional safety (Cogan et al., 2024). Similarly, Hyseni Duraku et al. (2024) examines mental health awareness, stigma, and help-seeking among Albanian university students across Western Balkan contexts, demonstrating how multi-layered social influences shape help-seeking rather than simple individual attitudes (Hyseni Duraku et al., 2024). These studies support a sociological interpretation where help-seeking is a norm-governed practice shaped by expectations about competence, reputation, confidentiality, and trust in institutions. In other words, the barrier is often not knowledge that support exists, but the moral and social risk of being seen as unable to cope within environments that reward stoicism and high output.

Bringing these strands together, the review strengthens a central sociological claim: healthy lifestyle culture in university settings is best understood as an emergent outcome of (i) institutional time regimes (workload, assessment, deadlines), (ii) digitally mediated availability norms, and (iii) moral-evaluative climates shaping stigma, legitimacy, and trust. The evidence indicates that students’ health practices are often rational adaptations to institutional demands rather than failures of self-control. This matters because many university health programs remain behavior-centric: they target sleep hygiene, stress management skills, or help-seeking encouragement while leaving the upstream conditions intact. The studies reviewed here suggest that such approaches risk limited effectiveness when they do not address how overload is normalized and reproduced through academic organization.

These findings justify why the present article needed to be written. A systematic (PRISMA-aligned) synthesis is valuable not because it “adds another paper on student stress,” but because it integrates fragmented literatures into an explanatory framework that can inform both sociological theory and higher education policy. The review clarifies how macro-level shifts—digitalization of learning, intensified performance measurement, and expanding expectations of self-management—translate into meso-level institutional routines and micro-level everyday practices. In this sense, the review contributes to sociology of education and youth by shifting the analytical center from “individual health behaviors” toward “institutional normativity and patterned adaptation.”

Beyond synthesizing existing evidence, the originality of the present review lies in developing an integrated sociological framework that connects institutional normativity, organizational structures, digitalization, and structural inequalities within a single analytical perspective. While previous studies have primarily examined specific health behaviors or evaluated individual interventions, this review demonstrates how these seemingly separate phenomena are interconnected through the institutional organization of university life. By reframing healthy lifestyle culture as a socially produced and institutionally embedded process rather than a collection of individual behaviors, the review provides a stronger theoretical foundation for future sociological research and for the development of university policies aimed at promoting sustainable student wellbeing.

Several institutional implications can be drawn from the reviewed evidence. First, interventions should be evaluated not only on individual outcomes (sleep hours, stress scores) but on whether they alter institutional pressures and norms: assessment clustering, workload transparency, response-time expectations, and the symbolic legitimacy of rest. Second, universities should treat digital availability norms as a governance issue: students’ sleep and recovery are compromised when learning platforms, groupwork systems, and academic communication function as 24/7 environments. Third, help-seeking initiatives require trust-building and confidentiality assurance, but also cultural change: stigma decreases when institutional practices communicate that seeking help is compatible with being a “good student,” not evidence of failure.

Finally, this review also highlights limitations in current research that future sociological work should address. Many empirical studies use cross-sectional designs and individual-level measures of stress and health, which can under-capture institutional causality in the formation and erosion of wellbeing-related competencies. The strongest contributions come from longitudinal designs and multi-wave analyses that link institutional exposures to health outcomes (e.g., workload → stress → health), as in Leshner et al. (2021), Yang et al. (2021) and Lin and Zhou (2022). Future work would benefit from combining institutional data (assessment calendars, digital platform activity patterns, policy rules) with student ethnographies and network analyses to map how norms circulate and stabilize. Such approaches would allow sociology to explain not only that stress and sleep deprivation are common, but how universities systematically produce cultures in which these conditions become normal.

Overall, the findings demonstrate that healthy lifestyle culture in universities should be understood as a product of institutional normativity rather than individual responsibility alone. Recognizing this shift has important implications for both sociological theory and university policy, emphasizing that sustainable student wellbeing depends not only on behavioral interventions but also on institutional environments that enable healthy everyday practices.

## 7 Conclusion

Healthy lifestyle culture among university students emerges as a socially structured phenomenon shaped by normative expectations, institutional arrangements, and culturally embedded interpretations of academic success. Student health-related practices are not isolated expressions of individual choice but are organized through shared meanings and routines that define what forms of conduct are considered legitimate within higher education. Universities, as central institutions in young people’s lives, play a decisive role in producing and stabilizing these norms through academic rhythms, performance expectations, and digitally mediated forms of engagement, thereby shaping students’ capacity to sustain wellbeing alongside academic participation.

A central conclusion of this review is that practices commonly framed as problematic—such as chronic stress, sleep deprivation, and delayed engagement with support services—are frequently normalized within university settings. These practices function as adaptive responses to institutional demands rather than deviations from healthy standards. Norms that valorize endurance, constant availability, and self-responsibility contribute to the moral regulation of student life, shaping how wellbeing is understood, managed, and often deferred in favor of academic performance. In this sense, universities do not merely influence student health outcomes but actively shape the boundaries of students’ self-regulatory and wellbeing-related competencies.

The analysis further indicates that healthy lifestyle culture is reproduced across multiple levels of university life. Peer interactions reinforce expectations of conformity, while institutional structures—assessment systems, workload organization, and digital infrastructures—embed these expectations into everyday routines. As a result, individual health practices reflect broader organizational logics that extend beyond campus life and influence future professional and social trajectories. From a sociological perspective, addressing student wellbeing therefore requires moving beyond behavior-focused interventions toward institutional and cultural change. Efforts to promote health that do not engage with the normative and structural conditions of academic life risk reinforcing individual responsibility narratives while leaving the sources of strain intact. Universities thus face the challenge of aligning educational objectives with conditions that support the sustainable development of wellbeing-related competencies as an integral part of academic participation rather than as external or compensatory activities.

More broadly, the formation of healthy lifestyle culture within universities carries significant social implications. As sites of socialization for emerging adults, universities contribute to shaping societal norms around productivity, self-care, and institutional trust. Understanding how health-related norms are produced in higher education provides critical insight into the reproduction of health inequalities and highlights the potential for universities to function as agents of social change. Recognizing healthy lifestyle culture as a collective and institutional phenomenon offers a foundation for developing more equitable, supportive, and health-promoting educational environments that enable students to sustain wellbeing across their educational trajectories and beyond.
